# Phagocytosis and nitric oxide production by peritoneal adherent cells in response to *Candida albicans* in aging: a collaboration to elucidate the pathogenesis of denture stomatitis

**DOI:** 10.1590/1678-7757-2016-0322

**Published:** 2017

**Authors:** Taiane Priscila GARDIZANI, Karen Henriette PINKE, Heliton Gustavo de LIMA, Vanessa Soares LARA

**Affiliations:** 1 Universidade de São Paulo, Faculdade de Odontologia de Bauru, Departamento de Cirurgia, Estomatologia, Patologia e Radiologia. Bauru, SP, Brasil.

**Keywords:** *Candida*-associated denture stomatitis, Immunosenescence, Peritoneal adherent cells, Phagocytosis, Nitric oxide

## Abstract

Elderly denture wearers are commonly affected by *Candida*-associated denture stomatitis (DS), an inflammatory process of the oral mucosa strongly associated with *Candida* spp and other microorganisms, as well as local and systemic factors. The impaired immune response against pathogens is among the inherent host factors that have been also associated with the pathogenesis of DS. Mononuclear phagocytes respond to the pathogens through phagocytosis followed by the production of several substances inside the phagosomes, among them are the reactive nitrogen species (RNS). A failure in these mechanisms may contribute to the DS development.

**Objective:**

The aim of this study was to investigate the influence of aging on the internalization and the production of nitric oxide (NO) by peritoneal adherent cells (PAC), in response to *Candida albicans* (*C. albicans*).

**Material and methods:**

PAC obtained from young and aged mice were challenged with dead or viable *C. albicans* by using predetermined proportions (cells:yeast) for 30 and 120 minutes. Phagocytosis was analyzed by acridine orange dye, and NO production by the Griess reaction.

**Results:**

*C. albicans* phagocytosis by PAC from aged mice was similar to that of young mice, although the cells from older mice cells present more internalized fungi compared with matched control. In addition, a tendency towards impaired NO production by peritoneal mononuclear phagocytes from aged mice was observed.

**Conclusions:**

PAC from aged mice may capture and store many fungi, which in turn may mean that these cells are effectively unable to eliminate fungi, probably due to impaired NO production. Therefore, considering the important role of *C. albicans* overgrowth in the pathogenesis of DS and the aspects observed in this study, aging may favor the onset and severity of local candidosis such as DS and its systemic forms.

## Introduction

*Candida*-associated denture stomatitis (DS) is among the most common types of local candidiasis, an oral lesion present in more than 60% of the denture-wearing individuals, with older persons being more frequently affected than the young population^14^. DS is strongly associated with the overgrowth of *Candida* spp on the inner denture surface, mainly consisting of *Candida albicans* (*C. albicans*), in addition to other local factors such as poor-fitting dentures and inadequate denture hygiene. Aspects inherent to the host are also strongly associated with DS, such as nutritional deficiencies, diabetes mellitus, use of immunosuppressive drugs and aging, since these factors contribute to a deficient immune response and hence to the establishment and maintenance of DS^14,25^.

*C. albicans* is a component of the normal microflora of the alimentary tract and mucocutaneous membranes of healthy humans. The host immune system is the major factor balancing the transition from commensalism to pathogenicity. When immune defenses are compromised, or the normal microflora balance is disrupted, species of *Candida* are transformed into opportunistic pathogenic killers that can invade the tissues^7,23^. In this scenario, epithelial cells and mononuclear phagocytes, such as resident macrophages, provide the first line of defense against *C. albicans* by the initial fungal recognition and production of mediators that recruit and activate neutrophils. Besides, macrophages exert anti-*Candida* activities by means of phagocytosis and ability to kill this fungus by producing various enzymes and toxic substances in the phagosomes^1,23,30^. After the establishment of specific immune responses against *Candida*, phagocytosis by macrophages is enhanced by interferon-gamma producing cells^23^. This Th-1 response seems to be crucial to the resolution of an oropharyngeal candidiasis model^9^. Among toxic substances present in phagolysosome are the reactive oxygen and nitrogen species (ROS and RNS, respectively). ROS are derived from the phagocyte membrane NADPH-oxidase system that generates free radicals (superoxide anion and hydroxyl radical), capable of reacting with other molecules and forming large amounts of hydrogen peroxide and superoxide by the “oxidative burst”. Nitric oxide (NO), another important reactive, is a RNS produced by the inducible nitric oxide synthase (iNOS), a cytosolic enzyme that is absent in resting macrophages^21,27^.

Nowadays, infections caused by *C. albicans* are a challenge to the development of effective treatments, partly due to mechanisms exerted by these fungi to escape the immunological response such as interference in the maturation of phagosomes, detoxification of the microbicidal compounds, and shielding of PAMPs^21,27^.

Another risk factor is biofilm formation. *C. albicans* commonly grows on abiotic or biological surfaces in association with other fungal species and bacteria. For fungi, biofilm formation provides the microbial community with protection and resistance to drugs^4^. DS is a more common type of oral candidiasis affecting denture wearers. The etiology and pathogenesis of DS are linked to biofilm formation by the *Candida* spp fungus. There are a high number of denture wearers among the older population, which, consequently, is severely affected by DS^16^.

Evidences suggest that aging has a significant influence on innate immunity, although age-related alterations in innate immune function are not necessarily equal to immunodeficiency, but rather to dysregulation of the immune response^20^. The increase in age leads to enhanced susceptibility to fungal, bacterial and viral infections, reactivation of latent viruses, and a decreased response to vaccines^15^. Furthermore, studies have suggested that a number of macrophage functions become compromised with advancing age, including chemotaxis, phagocytosis, ROS and RNS production, and induction of certain cytokine responses^19,28,29^. Thus, altered function of these cells due to aging may play a key role in the defense mechanisms, including infectious diseases caused by *C. albicans*, such as DS.

Therefore, the present study evaluated the phagocytic ability and NO production by *C. albicans-*stimulated peritoneal adherent cells (PAC) from aged mice compared with young mice after *in vitro* stimulation with dead or viable opsonized *C. albicans,* for 30 and 120 minutes.

## Material and Methods

### Isolation of peritoneal adherent cells

Young (2-3 months) and aged (18-20 months) C57BL/6 male mice were purchased from the Central Animal Facility of the Bauru School of Dentistry, University of São Paulo. All assays involving mice were approved by the Ethics Committee on Animal Research of the Bauru School of Dentistry, University of São Paulo (Permit No. 011/2010).

Peritoneal adherent cells (PAC) from young and aged mice were harvested by injecting and withdrawing 8 mL cell culture medium (RPMI 1640, Gibco^®^, Grand Island, New York, USA). The cells in both groups were diluted in a complete cell-culture medium (RPMI 1640 supplemented with 5% heat-inactivated fetal bovine serum and 1% penicillin/streptomycin) and plated at same density, i.e., 1x10^6^ cells, in a 24-well tissue culture plate containing a sterile glass coverslip, measuring 13 mm in diameter in each well. PAC were allowed to adhere to the glass surface at 37°C for 2 hours in a 5% CO_2_ atmosphere. Non-adherent cells were removed by washing and the mononuclear phagocytes (macrophages and B-1 lymphocytes)^2^ were cultured overnight before the challenge.

### *Candida albicans* ATCC 90028 – preparation and opsonization

*C. albicans* ATCC 90028 strains were obtained from the stock culture collection of the Integrated Research Center, Bauru School of Dentistry, University of São Paulo. Yeast cells were grown on Sabouraud dextrose broth (Difco) and were maintained at 28°C for 24 hours. Yeast cells were washed with Phosphate Buffered Saline (PBS, pH 7.4) and dilutions of the fungus were made in a simple culture medium (RPMI 1640) at different concentrations of viable and dead fungi, according to the phagocytosis assay. Dead fungi were killed by heat in an autoclave, at 121°C, for 30 minutes. One mL of viable and dead *C. albicans* ATCC 90028 yeasts was opsonized with 50 µL of human serum for 20 minutes in a shaking incubator (75 rpm) at 37°C, since the serum is rich in complement proteins irrespective of originating from different species.

### *In vitro* challenge of PAC with *Candida albicans*

PAC were challenge with dead or viable *C. albicans* by using predetermined proportions (cells:yeast), at 37°C in a 5% CO_2_ atmosphere, for 30 and 120 minutes. For dead yeasts, the proportions were 1:1 (*Ca*D1), 1:2 (*Ca*D2), or 2:1 (*Ca*D3); and for viable yeasts, 1:1 (*Ca*V1), 1:5 (*Ca*V2), or 5:1 (*Ca*V3), according to previous reports^21,26^. After the challenges, supernatants were centrifuged at 5000xg for 5 minutes to make them free of cells; and stored at -80°C for later nitric oxide (NO) determination by the Griess reaction. Cells and fungi adhered to the coverslips were fixed with 2% paraformaldehyde and stained with 0.05 mg/mL acridine orange dye. The coverslips were mounted with Permount^TM^ resin on glass slides and visualized by UV light. Presence or absence of *C. albicans* internalization by PAC (showing green or orange colors) was quantitatively assessed by using the fluorescence microscope Axiostar HBO plus 50/AC (Zeiss, Germany) at 100x magnification, from 25 consecutive fields. For a qualitative analysis, by differentiating internalized fungi from those on the surface of the PAC, a confocal laser scanning microscope was used (TCS model, SPE, Leica, Mannheim, Germany). The results were obtained from the average of three independent experiments, by using the total number of cells counted *per* coverslip (25 fields) in each experiment. Based on the mean of total cells, average percentages of cells with internalized fungi were obtained. Phagocytosis was also characterized by considering the number of yeast/budding and yeast/pseudohyphae within the cells (1, 2, 3, 4, 5, or more).

### Nitric oxide assay

The Griess reaction was applied to analyze nitrite formation. The supernatants without cells (100 µL) were mixed with the same volume of Griess reagent (N-naphthyl-ethylenediamine plus sulfanilamide) in a 96-well non-tissue culture plate and the absorbance was read at 540nm using an automated microplate reader (Synergy Mx Monochromator-Based Multi-Mode Microplate Reader, BioTek Instruments, USA). Results were determined by comparison with a standard curve performed with sodium nitrite in the following concentrations: 200, 100, 50, 25, 12.5, 6.25, 3.125, and 1.5 μM.

### Statistical analysis

Statistical analysis was performed by the variance test (ANOVA) followed by Fisher LSD test, at 95% confidence levels (Statistica 10 software), applied to evaluate the statistical significance of the results. All means were calculated from three independent experiments.

## Results

### Evaluation of PAC infected with *C. albicans*

**Figure 1:**
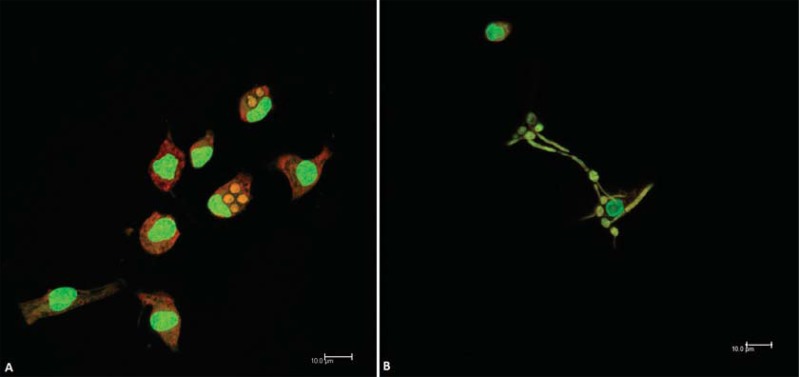
Internalization of dead (A) or viable (B) *C. albicans* by peritoneal adherent cells (PAC) from aged mice after 30 minutes, in the proportion 1:1 (*Ca*D1 and *Ca*V1, respectively)

After 30 and 120 minutes of challenge with different amounts of dead or viable yeast, the coverslips were stained with acridine orange. Both cells and *C. albicans* could be seen in green or orange-red color (Figure 1).

**Figure 2:**
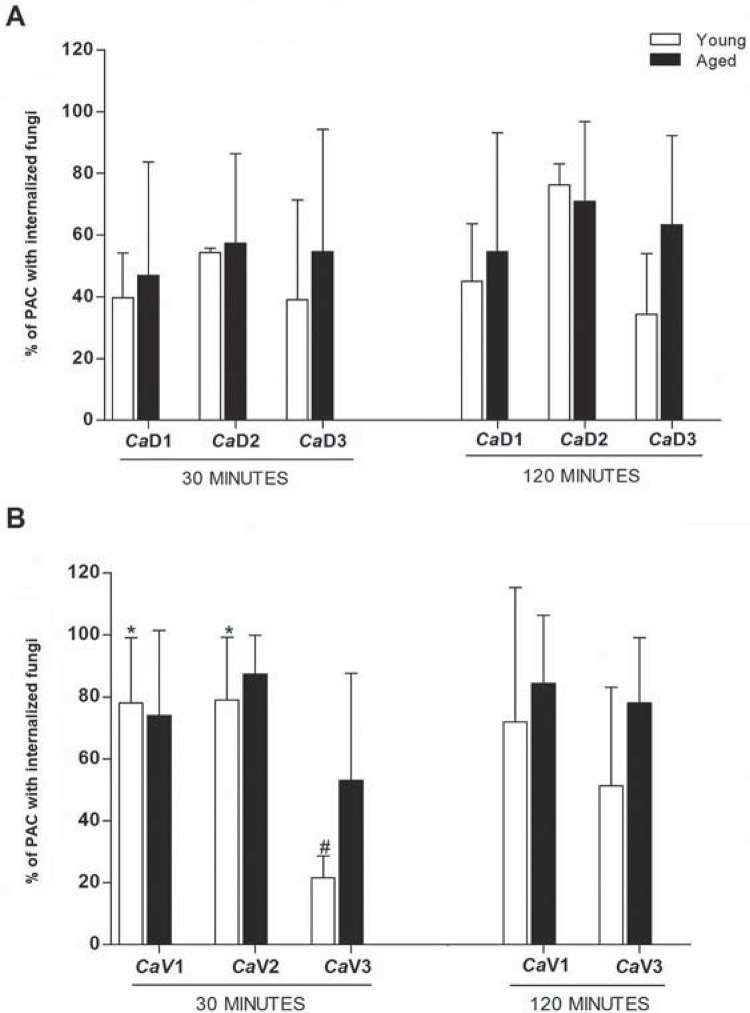
Percentage (mean) of peritoneal adherent cells (PAC) from young or aged mice, presenting internalized *C. albicans*, after challenge for 30 minutes (A) and 120 minutes (B) with different proportions of cells:dead fungi – 1:1 (*Ca*D1), 1:2 (*Ca*D2), 2:1 (*Ca*D3), or cells:viable fungi – 1:1 (*Ca*V1), 1:5 (*Ca*V2), 5:1 (*Ca*V3), from three independent experiments. Different symbols represent statistically significant differences among the proportions of cells:yeast for each age group (factorial ANOVA followed by Fisher LSD test; p<0.05). No statistical difference was found between age (old vs. young) groups

Irrespective of fungal viability (dead or viable), the percentage of PAC with *C. albicans* internalized from young donors was similar to values obtained from the aged group when each proportion and each period was individually considered. In addition, the percentage of peritoneal cells phagocyting *C. albicans* was not time-dependent, since there were no statistically significant differences between the two periods analyzed, when each proportion and each group was individually considered (Figure 2).

Although without any statistic difference, the assays performed with more dead fungal cells than peritoneal cells (1:2 – *Ca*D2) resulted in increased percentage of phagocytosis, particularly in the young group. This suggests that the phagocytic capacity of PAC is proportional to availability of dead yeast.

Considering the phagocytosis of viable yeast, the greatest differences were observed at 30 minutes, since the proportion *CaV*2 at 120 minutes could not be counted. In the young group, the proportions *Ca*V1 and *Ca*V2 resulted in a significant percentage of cells with internalized fungi, varying between 79% and 87%. PAC from aged group also showed high percentages of phagocytosis in the proportions *Ca*V1 and *Ca*V2, but without statistically significant difference.

As previously mentioned, the higher proportion of viable fungi:to cell (1:5 - *Ca*V2) made it impossible to count the phagocytes at 120 minutes. In this period, we observed remnants of peritoneal mononuclear phagocytes with many budding yeasts and pseudohyphae, suggesting that the survival and proliferation mechanisms of *C. albicans* outweighed the defense of PAC that succumbed and died.

The quantitative evaluation of the number of dead or viable internalized yeasts (1, 2, 3, 4, 5, or more) by PAC was also performed. In assays with dead fungi, the majority of peritoneal mononuclear phagocytes from the young group contained two yeasts within them, irrespective of the fungal proportions and the time of phagocytosis (p<0.01). On the other hand, PAC from aged animals showed higher percentages of many internalized dead fungi (five or more), mainly after 120 minutes compared with the matched young animals. This difference was statically significant (p<0.01) in the proportion of *Ca*D2 (Table 1).

**Table 1:** Percentage (mean) of peritoneal adherent cells (PAC), derived from young or aged mice, presenting yeast/budding yeast/pseudohyphae of internalized *C. albicans* (1, 2, 3, 4, 5, or more) after challenge with different proportions of cell:dead/viable fungi for 30 minutes (first data) and 120 minutes (secondary data), from three independent experiments. Underlined number represents statistically significant difference between young and aged group. Different symbols represent statistically significant difference for each row and its matched column

Proportions PAC: *C. albicans*	PAC from young mice with different amounts of phagocytosed *C. albicans* (%)				
	1	2	3	4	5/+
*Ca*D					
1 (1:1)	32* – **26**	36* – 38*	*18* – **19**	*09*# – **09**#	*06*# – **08**#
2 (1:2)	*19* – **21**	35* – 39*	*23* – **21**	*11*# – **13**#	*11*# – **06**#
3 (2:1)	*27* – 39*	36* – 38*	*15* – **13**#	*12*# – **05**#	*11*# – **05**#
*Ca*V					
1 (1:1)	*34* – **51**	*30* – **26**	*12* – **14**	*14* – **06**	*11* – **04**
2 (1:5)	*23* – °°	*22* – °°	*17* – °°	*13* – °°	*24* – °°
3 (5:1)	57* – 65*	*34* – **24**	*05* – **7**#	*00*# – **03**#	*04*# – **01**#
					

**Proportions PAC: *C. albicans***	**PAC from aged mice with different amounts of phagocytosed *C. albicans* (%)**				

	1	2	3	4	5/+
*Ca*D					
1 (1:1)	35* – **21**	*30* – **30**	*12*# – **10**	*08*# – **09**	*15* – **30**
2 (1:2)	*24* – **26**	35* – **21**	*15* – **12**	*10*# – **09**	*17* – **32**
3 (2:1)	*30* – **25**	*26* – **28**	*16* – **11**	*09* – **13**	*19* – **23**
*Ca*V					
1 (1:1)	*34* – **30**	*29* – **24**	*20* – **18**	*08* – **14**	*09* – **14**
2 (1:5)	*21* – °°	*22* – °°	*17* – °°	*13* – °°	*26* – °°
3 (5:1)	*46* – **33**	*22* – **26**	*09* – **12**	*07* – **10**	*16* – **18**

°°The high number of budding yeasts and pseudohyphae of *C. albicans* or remnants of mononuclear cells did not allow cell counting.

Higher percentages of PAC with 1 or 2 viable internalized yeast/budding yeast/pseudohyphae were observed in the young group, except in the proportion of *Ca*V2. Particularly in the proportion of *Ca*V3, the percentage of PAC with one viable yeast/budding yeast/pseudohyphae within them were statically higher than those representing cells with 4, 5, or more internalized *C. albicans* (p<0.01). In the same analysis, no difference was observed in the assays with viable *C. albicans* and PAC from aged groups (Table 1).

It is noteworthy that in the proportion of 1:5 (*Ca*V2), after 120 minutes, PAC derived from both young and aged animals were covered by large amounts of budding yeasts and pseudohyphae of *C. albicans*. This high number of *C. albicans* associated with remnants of peritoneal mononuclear cells did not allow the cell counting, as previously reported.

### Determination of nitric oxide (NO) production

**Figure 3:**
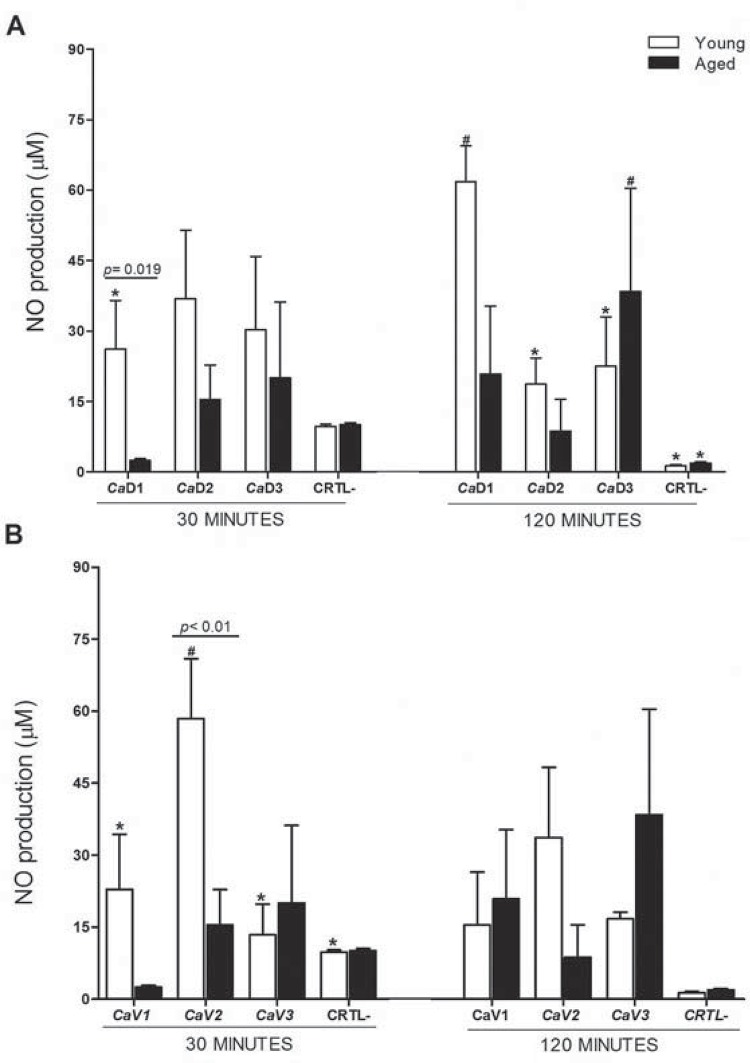
Mean production of NO (µM) by peritoneal adherent cells (PAC) from young and aged animals, after 30 and 120 minutes of challenge with different proportions of cells compared with dead *C. albicans* (A) – 1:1 (*Ca*D1), 1:2 (*Ca*D2), 2:1 (*Ca*D3), and cells compared with viable *C. albicans* (B) – 1:1 (*Ca*V1), 1:5 (*Ca*V2), 5:1 (*Ca*V3), from three independent experiments. CTRL- represents NO values from macrophages without stimulation. Different symbols represent statistically significant differences among the proportions of cells:yeast for each age group (factorial ANOVA followed by Fisher LSD test; p<0.05)

In general, except in the proportions of low fungal burden, PAC from older animals showed a tendency to produce less NO than the cells from young animals. This production was generally maintained over time. The difference in NO production between aged and young PAC was statistically significant in the proportions *Ca*D1 (Figure 3A) and *Ca*V2 (Figure 3B), at 30 minutes (p=0.019 and p<0.01, respectively). The aforementioned trend was not observed in unstimulated cells, suggesting greater activation by PAC derived from young mice in trying to kill the fungi than those from aged mice.

A time-dependent increase in NO production was observed after PAC from young mice were stimulated with dead *C. albicans* (*Ca*D1) (p=0.019). Furthermore, the different proportions caused significant alterations in NO production by PAC from young mice after 120 and 30 minutes of stimulation with dead and viable fungi, respectively (Figure 3).

## Discussion

The role of innate immunity against infections in older persons is divergent. Some authors believe that the cells responsible for primary response are altered with aging^17,18,20,22,28^. Variable results were also obtained concerning the impact of aging on phagocytosis in mice. This function has been reported to be regulated negatively^18^, positively^17^, or even unchanged^22^ with advancing age.

The present *in vitro* study with dead or viable *C. albicans* showed that PAC from aged animals exhibited a similar percentage of phagocytosis as that from young mice. These results are in agreement with studies that showed that the expression of important macrophage receptors, involved either directly or indirectly in the recognition of microorganisms, such as CD14, CD11b, CD18, CD36, Toll-like receptors 1, 2 and 4 (TLR1, TLR2 e TLR4), mannose receptor (CD206), dectin-1, and scavenger receptor, are maintained in aging individuals^18,20^. Thus, at first, these results led us to reasoning that the phagocytic capacity of mononuclear phagocytes from senescent animals would be well preserved.

However, in the group of older animals, after 120 minutes of challenge, the percentage of PAC with many internalized fungi (five or more), in comparison with cells presenting few fungi inside them (less than five), was higher than those found in the young, regardless of the cell/yeast proportion or viability of the fungi. Possibly, PAC from aged mice are storing these fungi that were successfully internalized due to their inability to kill and eliminate it. Furthermore, these cells could serve as disseminator vehicles, promoting dispersion of the pathogen in the body and protecting the fungus from other mechanisms of the innate immune response^3,30^.

In addition, we observed that cells from aged animals showed a tendency towards lower NO production than those of young animals. Altogether, these results suggested that the PAC from older animals may fail to kill internalized *C. albicans,* probably due to impaired NO production, since Elahi, et al.^8^ (2001) showed the correlation between decreased NO production in saliva and increased viability of *C. albicans*. The findings of the present study corroborate the view that age is a contributing factor to functional impairment of innate immunity, representing a strong influence on susceptibility to *Candida* diseases, as highlighted in numerous studies^17,18,20,22,28^. Particularly, advancing age has been considered one of the predisposing factors to *Candida* infections in denture wearers^12,13,16^.

Indeed, the development of candidiasis in humans appears to pose higher risk with advancing age^25^, since immune mechanisms seem to be altered, including those related to the production of nitric oxide; however, studies in this context have provided conflicting results. Researchers have shown that iNOS, as well as the production of nitrite and other intermediaries, are impaired in senescent mice when stimulated by a variety of agents^19,29^. Conversely, the increased production of iNOS mRNA and nitrite by thioglycolate-elicited peritoneal macrophages from aged mice has also been reported after stimulus with LPS when compared with those of young animals^5^.

In addition, *C. albicans* itself presents the ability to escape from the microbicidal mechanisms of phagocytosis, including the potential to detoxify the phagosome, adapting to environments with microbicidal compounds, such as nitric oxide^6,10,11,27^. It is possible that the suggested failure of PAC performance in aged animals, observed in the present study, resulted from mechanisms for subversion by the internalized fungus in the immune system.

In denture wearers, the *C. albicans* capacity of biofilm formation on denture surfaces exposes the oral mucosa of these subjects to a continuous fungal load. Considering subjects with DS, the more common oral candidiasis, the communities found in dentures are usually formed by different *Candida* spp, mainly *C. albicans*, and various bacteria^4,16^. This association causes increased expression of virulence genes and those associated with epithelial damage and invasion^4^.

Following this line of reasoning, age is a really key aspect in the development and maintenance of DS. We also demonstrated impairment in the function of human salivary or blood neutrophils^12,13^ and monocytes^24^ obtained from volunteers with DS compared with denture wearers without this disease.

## Conclusions

Considering the pathogenesis of DS and the findings about the increased susceptibility of older persons to infections, the results of the present study reinforce the possibility of impairment of the innate immune response in aging, possibly influencing the response to *C. albicans* including localized diseases such as DS. In this context, in the older population (more frequently affected by DS) the failure of mononuclear phagocytes to kill fungi may represent a contributory factor to the development of DS.
