# ERRATUM

**DOI:** 10.1590/1678-7757-2017er002

**Published:** 2017

**This correct the article: 10.1590/1678-7757-2016-0322**

Due to a publishing error the article “Phagocytosis and nitric oxide production by peritoneal adherent cells in response to Candida albicans in aging: a collaboration to elucidate the pathogenesis of denture stomatitis”, published at Journal of Applied Oral Science 25(3):265-73 was printed with the following errors:

**p. 270**

**Where you read:** [Table t1]

**Table 1 t1:** Percentage (mean) of peritoneal adherent cells (PAC), derived from young or aged mice, presenting yeast/budding yeast/pseudohyphae of internalized *C. albicans* (1, 2, 3, 4, 5, or more) after challenge with different proportions of cell:dead/viable fungi for 30 minutes (first data) and 120 minutes (secondary data), from three independent experiments. Underlined number represents statistically significant difference between young and aged group. Different symbols represent statistically significant difference for each row and its matched column

Proportions PAC: *C. albicans*	PAC from young mice with different amounts of phagocytosed *C. albicans* (%)				
	1	2	3	4	5/+
*Ca* D					
1 (1:1)	*32* * – **26**	*36* * – **38** *	*18* – **19**	*09* # – **09** #	*06* # – **08** #
2 (1:2)	*19* – **21**	*35* * – **39** *	*23* – **21**	*11* # – **13** #	*11* # – **06** #
3 (2:1)	*27* – **39** *	*36* * – **38** *	*15* – **13** #	*12* # – **05** #	*11* # – **05** #
*Ca* V					
1 (1:1)	*34* – **51**	*30* – **26**	*12* – **14**	*14* – **06**	*11* – **04**
2 (1:5)	*23* – °°	*22* – °°	*17* – °°	*13* – °°	*24* – °°
3 (5:1)	*57* * – **65** *	*34* – **24**	*05* – **7** #	*00* # – **03** #	*04* # – **01** #
					
**Proportions PAC: *C. albicans***	**PAC from aged mice with different amounts of phagocytosed *C. albicans* (%)**				
	1	2	3	4	5/+
*Ca* D					
1 (1:1)	*35* * – **21**	*30* – **30**	*12* # – **10**	*08* # – **09**	*15* – **30**
2 (1:2)	*24* – **26**	*35* * – **21**	*15* – **12**	*10* # – **09**	*17* – **32**
3 (2:1)	*30* – **25**	*26* – **28**	*16* – **11**	*09* – **13**	*19* – **23**
*Ca* V					
1 (1:1)	*34* – **30**	*29* – **24**	*20* – **18**	*08* – **14**	*09* – **14**
2 (1:5)	*21* – °°	*22* – °°	*17* – °°	*13* – °°	*26* – °°
3 (5:1)	*46* – **33**	*22* – **26**	*09* – **12**	*07* – **10**	*16* – **18**

°°The high number of budding yeasts and pseudohyphae of *C. albicans* or remnants of mononuclear cells did not allow cell counting.

**Should read:** [Table t2]

**Table 1 t2:** Percentage (mean) of peritoneal adherent cells (PAC), derived from young or aged mice, presenting yeast/budding yeast/pseudohyphae of internalized *C. albicans* (1, 2, 3, 4, 5, or more) after challenge with different proportions of cell:dead/viable fungi for 30 minutes (italic) and 120 minutes (bold), from three independent experiments. Underlined number represents statistically significant difference between young and aged group. Different symbols represent statistically significant difference for each row and its matched column

Proportions PAC: *C. albicans*	PAC from young mice with different amounts of phagocytosed *C. albicans* (%)				
	1	2	3	4	5/+
*Ca* D					
1 (1:1)	*32* * – **26**	*36* * – **38** *	*18* – **19**	*09* # – **09** #	*06* # – **08** #
2 (1:2)	*19* – **21**	*35* * – **39** *	*23* – **21**	*11* # – **13** #	*11* # – **06** #
3 (2:1)	*27* – **39** *	*36* * – **38** *	*15* – **13** #	*12* # – **05** #	*11* # – **05** #
*Ca* V					
1 (1:1)	*34* – **51**	*30* – **26**	*12* – **14**	*14* – **06**	*11* – **04**
2 (1:5)	*23* – °°	*22* – °°	*17* – °°	*13* – °°	*24* – °°
3 (5:1)	*57* * – **65** *	*34* – **24**	*05* – **07** #	*00* # – **03** #	*04* # – **01** #
					
**Proportions PAC: *C. albicans***	**PAC from aged mice with different amounts of phagocytosed *C. albicans* (%)**				
	1	2	3	4	5/+
*Ca* D					
1 (1:1)	*35* * – **21**	*30* – **30**	*12* # – **10**	*08* # – **09**	*15* – **30**
2 (1:2)	*24* – **26**	*35* * – **21**	*15* – **12**	*10* # – **09**	*17* – **32**
3 (2:1)	*30* – **25**	*26* – **28**	*16* – **11**	*09* – **13**	*19* – **23**
*Ca* V					
1 (1:1)	*34* – **30**	*29* – **24**	*20* – **18**	*08* – **14**	*09* – **14**
2 (1:5)	*21* – °°	*22* – °°	*17* – °°	*13* – °°	*26* – °°
3 (5:1)	*46* – **33**	*22* – **26**	*09* – **12**	*07* – **10**	*16* – **18**

°°The high number of budding yeasts and pseudohyphae of *C. albicans* or remnants of mononuclear cells did not allow cell counting.

**p. 271**

**Where you read:**

**Figure 3-** Mean production of NO (µM) by peritoneal adherent cells (PAC) from young and aged animals, after 30 and 120 minutes of challenge with different proportions of cells compared with dead *C. albicans* (A) – 1:1 ( *Ca* D1), 1:2 ( *Ca* D2), 2:1 ( *Ca* D3), and cells compared with viable *C. albicans* (B) – 1:1 ( *Ca* V1), 1:5 ( *Ca* V2), 5:1 ( *Ca* V3), from three independent experiments. CTRL- represents NO values from macrophages without stimulation. Different symbols represent statistically significant differences among the proportions of cells:yeast for each age group (factorial ANOVA followed by Fisher LSD test; p<0.05)

**Should read:**

**Figure 3-** Mean production of NO (µM) by peritoneal adherent cells (PAC) from young and aged animals, after 30 and 120 minutes of challenge with different proportions of cells compared with dead *C. albicans* (A) – 1:1 ( *Ca* D1), 1:2 ( *Ca* D2), 2:1 ( *Ca* D3), and cells compared with viable *C. albicans* (B) – 1:1 ( *Ca* V1), 1:5 ( *Ca* V2), 5:1 ( *Ca* V3), from three independent experiments. CTRL- represents NO values from PAC without stimulation. Different symbols represent statistically significant differences among the proportions of cells:yeast for each age group (factorial ANOVA followed by Fisher LSD test; p<0.05)

